# Dimorphic effect of TFE3 in determining mitochondrial and lysosomal content in muscle following denervation

**DOI:** 10.1186/s13395-024-00339-1

**Published:** 2024-04-20

**Authors:** Ashley N. Oliveira, Jonathan M. Memme, Jenna Wong, David A. Hood

**Affiliations:** School of Kinesiology and Health Science Muscle Health Research Centre, York University, 4700 Keele St, Toronto, ON M3J 1P3 Canada

**Keywords:** Mitochondrial respiration, ROS emission, Sex differences, Lysosomal biogenesis, Mitophagy, Autophagy

## Abstract

**Background:**

Muscle atrophy is a common consequence of the loss of innervation and is accompanied by mitochondrial dysfunction. Mitophagy is the adaptive process through which damaged mitochondria are removed via the lysosomes, which are regulated in part by the transcription factor TFE3. The role of lysosomes and TFE3 are poorly understood in muscle atrophy, and the effect of biological sex is widely underreported.

**Methods:**

Wild-type (WT) mice, along with mice lacking TFE3 (KO), a transcriptional regulator of lysosomal and autophagy-related genes, were subjected to unilateral sciatic nerve denervation for up to 7 days, while the contralateral limb was sham-operated and served as an internal control. A subset of animals was treated with colchicine to capture mitophagy flux.

**Results:**

WT females exhibited elevated oxygen consumption rates during active respiratory states compared to males, however this was blunted in the absence of TFE3. Females exhibited higher mitophagy flux rates and greater lysosomal content basally compared to males that was independent of TFE3 expression. Following denervation, female mice exhibited less muscle atrophy compared to male counterparts. Intriguingly, this sex-dependent muscle sparing was lost in the absence of TFE3. Denervation resulted in 45% and 27% losses of mitochondrial content in WT and KO males respectively, however females were completely protected against this decline. Decreases in mitochondrial function were more severe in WT females compared to males following denervation, as ROS emission was 2.4-fold higher. In response to denervation, LC3-II mitophagy flux was reduced by 44% in females, likely contributing to the maintenance of mitochondrial content and elevated ROS emission, however this response was dysregulated in the absence of TFE3. While both males and females exhibited increased lysosomal content following denervation, this response was augmented in females in a TFE3-dependent manner.

**Conclusions:**

Females have higher lysosomal content and mitophagy flux basally compared to males, likely contributing to the improved mitochondrial phenotype. Denervation-induced mitochondrial adaptations were sexually dimorphic, as females preferentially preserve content at the expense of function, while males display a tendency to maintain mitochondrial function. Our data illustrate that TFE3 is vital for the sex-dependent differences in mitochondrial function, and in determining the denervation-induced atrophy phenotype.

**Supplementary Information:**

The online version contains supplementary material available at 10.1186/s13395-024-00339-1.

## Introduction

Skeletal muscle mass loss (i.e. atrophy) is a consequence of denervation, and is also commonly seen with prolonged muscle disuse, aging and cancer [1], the severity of which is a strong predictor of patient outcome and mortality [2]. There remains a gap in the literature regarding how biological sex influences the mechanisms involved in atrophy. This is problematic as it has been documented that females are more prone to developing muscle weakness during hospital visits requiring intensive care and have a higher incidence of mortality [3]. Therefore, there is an urgent need to better understand muscle atrophy in female subjects.

Atrophy is often preceded by aberrant mitochondrial phenotypes [4–6]. Declines in mitochondrial enzymatic activity, reductions in coupling efficiency, impaired ATP production, and decrements in oxygen consumption rates are observed with prolonged disuse such as in models of hindlimb unloading, immobilization, mechanical ventilation and denervation [1, 7–14]. Elevations in reactive oxygen species (ROS) have also been seen and are thought to be a major driver in the muscle atrophy phenotype [15–17]. Recently, it was shown that despite males having higher ROS emission basally, the induction of ROS production following hindlimb unloading is greater in females [18, 19].

Due to the exacerbated mitochondrial dysfunction observed during denervation, there is a greater cellular requirement for mitophagy, the selective removal of damaged mitochondria via the lysosomes [20–22]. Dysfunctional mitochondria are segregated from the reticulum through events of fission and targeted for degradation [23–26]. A variety of mitophagic signaling pathways have been described, which all culminate in engulfment by the autophagosome and trafficking to the lysosome [24, 27–29]. The lysosomes are critical sites for catabolism, and are tightly linked to mitochondrial health and cellular homeostasis [30]. Lysosomes are regulated by the microphthalamia (MiT) family of transcription factors, namely TFEB and TFE3, which have been shown to respond to various signals including nutrient deprivation, ROS and Ca^2+^ influx [31–36]. While considerable effort has been devoted to the study of TFEB, much less is known about the impact of TFE3 on lysosome formation.

Increases in the expression of lysosomal markers are seen with denervation, as are the regulators TFEB and TFE3, indicating a greater need for lysosomal activity [37]. Recently, reductions in mitophagy flux and increased inclusions found within skeletal muscle were observed in male rats following denervation. These inclusions are thought to be indicative of lysosomal dysfunction [21]. Basally there is evidence to suggest that females have increased autophagy machinery and lysosomal content in comparison to males [37, 38]. This would indicate a better maintenance of mitochondria during an imposed stress such as disuse, however some data suggest a worse mitochondrial phenotype [19]. Increased LC3-II and p62 levels have been found in female rodents following hindlimb unloading in comparison to males [19] but formal flux measurements have yet to be done. The purpose of this study was to investigate the role of the lysosomal transcriptional regulator TFE3 on the trajectory of muscle loss and the mechanisms of mitochondrial functional impairments with denervation, since this condition may present divergent responses compared to models of disuse. Since the influence of sex has been proposed in the literature but largely unexplored, analyses were conducted in both males and females to capture sex differences in atrophic conditions. We hypothesized that females would have some mitophagic blockade or impairment leading to a worse denervation-induced mitochondrial phenotype. Similarly, we speculated that TFE3 KO animals would exhibit a paradoxical phenotype whereby mitochondria were preserved due to lysosomal impairments, but that the functionality of these organelles would be poor.

## Methods

### Animal handling and sciatic denervation surgery

C57BL/6J (WT) and B6.129S1-*Tfe3*^*tm − 1Est*^*/*Mmjax were obtained from Jackson Laboratories and have been used previously [39]. Three month old male and females animals (*n* = 7–16 per group) were subjected to sciatic nerve denervation surgery for up to 7 days [7]. During deep anaesthetized using isofluorane, a 2–3 mm section of the sciatic nerve was excised on one hindlimb, while the other was sham-operated serving as an internal control. Animals were given analgesics (Meloxicam, 2 µg/g body weight on the day of surgery, and 1 µg/g body weight on the subsequent day) and antibiotics (Baytril, 5 mg/kg) post-surgery and provided food and water *ad libitum* throughout the treatment. Animals were individually housed following surgery and kept in 12 h light/dark cycle. The affected hindlimb muscles were collected for analysis under deep anaesthesia using isoflurane and were sacrificed via cervical dislocation. All procedures were approved by the Animal Care Committee at York University under the Canadian Council of Animal Care.

### Colchicine treatment and flux measurements

A subset of animals was treated with either colchicine (0.4 mg/kg/day; Col; C9754, Sigma) or 0.9% saline as vehicle (Veh) via intraperitoneal injection for 3 days prior to tissue collection as previously described [21, 40]. Constituents of the autophagosome were measured via immunoblotting in both mitochondrial fractions and whole muscle lysates to accurately capture mitophagy and autophagy. To calculate flux, the Veh values were subtracted from the mean Col value for the corresponding timepoint.

### Cytochrome C oxidase (COX) activity

COX activity was used as an indication of mitochondrial content. Briefly, frozen portions of TA muscle were lysed in enzyme extraction buffer using the Qiagen TissueLyser II and sonicated (3 × 3 s, at 30% power). A buffered test solution with fully reduced horse heart cytochrome c (C-2506, Sigma) was prepared and combined with enzyme extracts in a 96-well plate. The maximal oxidation rate of fully reduced cytochrome c was measured by the absorbance at 550 nm at 30 °C using a microplate reader (Cytation 5, BioTek).

### Mitochondrial fractionation

The gastrocnemius muscle was minced on a chilled watch glass on ice immediately after collection. Mitochondrial isolation buffer (67mM sucrose, 50mM Tris, 50mM KCl, 10mM EDTA, 0.2% BSA; pH 7.4) was added to the minced tissue, homogenized and centrifuged at 1,200 xg for 15 min at 4 °C. Following this, the supernate was centrifuged at 12,000 g to pellet mitochondria. The supernate was removed and the sample was resuspended again, centrifuged at 12,000 g for 20 min, followed by a final resuspension.

### High-resolution respirometry and ROS emission

High-resolution respirometry (Oxygraph-2 K, Oroboros Instruments) was performed on a medial section of the control and denervated TA. As previously described [7], fibers were mechanically teased apart in ice-cold BIOPS (2.77mM CaK_2_EGTA, 7.23mM K_2_EGTA, 7.55mM Na_2_ATP, 6.56mM MgCl_2_⋅6H_2_O, 20mM taurine, 15mM Na_2_ phosphocreatine, 20mM imidazole, 0.5mM DTT, 50 mM 2-(*N*-morpholino)ethanesulfonic acid hydrate, and pH 7.1), and permeabilized in BIOPS containing 40 µg/µl saponin at 4° C for 30 min and washed twice with Buffer Z (105mM K-2-(*N*-morpholino)ethanesulfonic acid, 30mM KCl, 10mM KH_2_PO_4_, 5mM MgCl_2_⋅6H_2_O, 1mM EGTA, 5 mg/ml bovine serum albumin, and pH 7.4). In the chamber, bundles of fibers (roughly 2-3 mg) were incubated with oxygenated Buffer Z supplemented with 10µM Amplex-Red(A36006, ThermoFisher) to measure ROS emission, 1µM Blebbistatin(B592500, Toronto Research Chemicals) to prevent tetanus of fiber and 25 U/ml Cu/Zn SOD1 to convert O_2_^−^ to H_2_O_2_ and 2mM EGTA. Substrates were subsequently titrated in the following order: pyruvate-malate to assess complex I supported respiration, ADP to assess complex I active respiration, and succinate to assess complex I + II active respiration. Respiration was normalized to background oxygen consumption rates and corrected for muscle wet weight.

### Protein extracts and immunoblotting

The distal portion of the TA was lysed in Sakamoto buffer (20mM Hepes, 2mM EGTA, 1% Triton X-100, 10% glycerol, 50mM β-glycerophosphate, 1mM PMSF, 1mM DTT) supplemented with phosphatase and protease inhibitors (11-697-498-001, Roche; P0044, Sigma; P5726, Sigma) using the Qiagen TissueLyser II and centrifuged at 12,000 xg for 10 min. Supernates were collected and protein concentration was measured using the Bradford method. Whole cell protein extracts or mitochondrial fractions (20 µg) were applied to a 10–18% SDS-PAGE gels and subsequently transferred onto a nitrocellulose membrane. Membranes were blocked in 5% skim milk in TBS-T (100mM TRIS, 100mM NaCl, 0.1% Tween 20) for 1 h at room temperature with constant agitation. Primary antibodies were diluted in 5% skim milk in TBS-T and incubated overnight at 4 °C and then horseradish-peroxidase (HRP)-linked secondary antibodies were applied the following day for 1 h at room temperature (See Table 1 for a list of antibodies). Blots were then imaged using ECL (1,705,061, BioRad) and iBright imager (iBright 1500, Invitrogen).

**Table 1:** List of antibodies used for western blotting

Protein	Concentration	Company	Catalogue Number
ATG7	1:1000	Sigma-Aldrich	A2856
Beclin1	1:1000	Cell Signaling Technology	3738 S
Cathepsin B	1:1000	Cell Signaling Technology	31,718 S
Cathepsin D	1:1000	Santa Cruz Biotechnology	SC-377,299
LC3	1:500	Cell Signaling Technology	4108 S
MCOLN1	1:1000	Invitrogen	PA1-46474
Parkin	1:1000	Cell Signaling Technology	4211 S
TFEB	1:500	MyBioSource	MBS120432
TFE3	1:500	Sigma-Aldrich	HPA023881
v-ATPase	1:1000	Santa Cruz Biotechnology	SC-55,544
HRP-linked Anti-mouse	As per manufacturer’s suggestions	Cell Signaling Technology	7074 S
HRP-linked Anti-rabbit	As per manufacturer’s suggestions	Cell Signaling Technology	7076 S

### Statistical analyses

Data presented are means ± SEM and statistical analyses were performed using Prism 10 (GraphPad Software). Two-way ANOVAs were conducted with repeated measures and Tukey post-hoc tests to assess the effect of genotype and sex basally, and to assess the effect of denervation and genotype within a particular sex. P values less than or equal to 0.05 were accepted as significant.

## Results

### Females are modestly spared from denervation-induced atrophy, as are male mice lacking TFE3

**Fig. 1:**
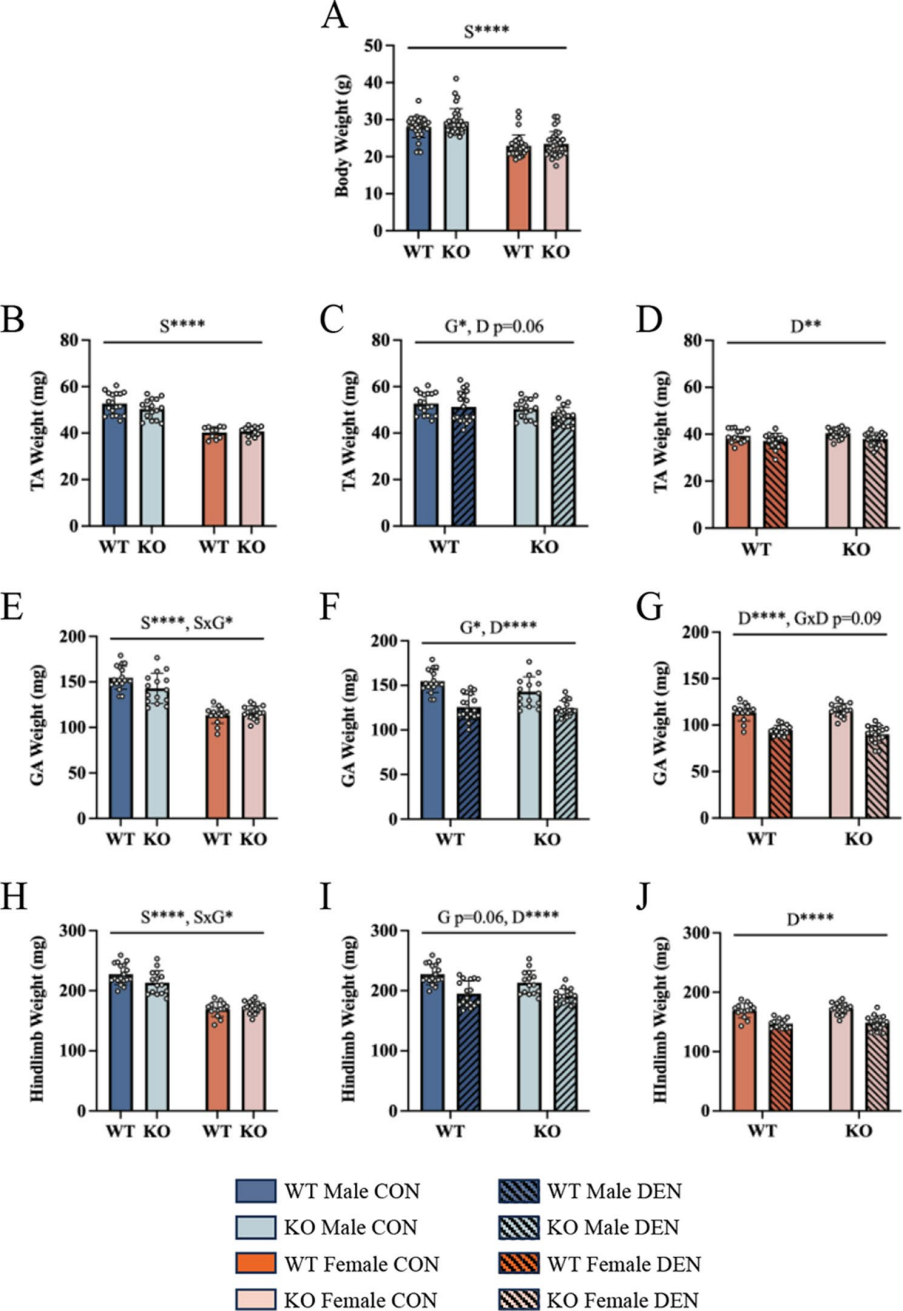
Females are modestly spared from denervation-induced atrophy, as are mice lacking TFE3. TA muscle weights basally stratified by sex (**A**). TA muscle mass following 7 days of denervation in both WT and KO males (**B**) and females (**C**). Gastrocnemius muscle weights basally stratified by sex (**D**). Gastrocnemius muscle mass following denervation in both WT and KO males (**E**) and females (**F**). Sum of hindlimb muscle weights, including TA, EDL, gastrocnemius, and soleus, in WT and KO animals stratified by sex basally (**G**). Sum of hindlimb muscles weights following denervation in both WT and KO males (**H**) and females (**I**). Absolute hindlimb muscle mass lost following denervation (**J**). Two-way ANOVAs were conducted with repeated measures between CON and DEN conditions, followed by Tukey post-hoc analyses. GA, gastrocnemius; G, denotes a main effect of genotype; S, denotes a main effect of sex; D, indicates a main effect of denervation; SxG, represents an interaction between genotype and sex; GxD, denotes an interaction between genotype and denervation; *, *p* < 0.05; **, *p* < 0.01; ****, *p* < 0.0001; *n* = 16

Phenotypically, TFE3 KO animals were not different from wildtype animals evidenced by similar body weights, and females of both genotypes were about 20% smaller than their male counterparts (Fig. 1A). Similarly, females had smaller TA and gastrocnemius muscle masses irrespective of genotype (Fig. 1B and E). However, gastrocnemius mass was modestly lower by 8% in TFE3 KO males in comparison to WT males, while no differences were seen in the females (Fig. 1E). This male-specific effect was also observed when all hindlimb muscle weights were pooled (Fig. 1H). As body weight did not differ between the genotypes, this reduced muscle mass observed in the males is likely countered by an increase in adiposity, as reported previously [39]. Notably, our data suggest that females lacking TFE3 do not share this phenotype, as muscle mass and body weight did not differ (Fig. 1A, B, E and G). This suggests that TFE3 is involved in the maintenance of skeletal muscle mass basally in a sex-dependent manner.

Following 7 days of denervation, significant muscle atrophy occurred across individual muscle groups including the TA (Fig. 1D) and gastrocnemius (Fig. 1F and G) and this was further exemplified when the sum of the hindlimb muscles was calculated (Fig. 1I and J). Interestingly, WT females appear to be mildly protected against denervation-induced atrophy (e.g. TA muscle) as these animals lost about 22 mg of total hindlimb muscle in comparison to WT males that lost about 34 mg (Fig. S1). Considering the sex- dependent differences in starting muscle mass, this represented a decline of 15% for the males, but only 12% for the females (Fig. S1). The absence of TFE3 did not have a significant impact on denervation-induced muscle mass loss, independent of sex.

### Sex-dependent response in mitochondrial content and function to denervation

**Fig. 2:**
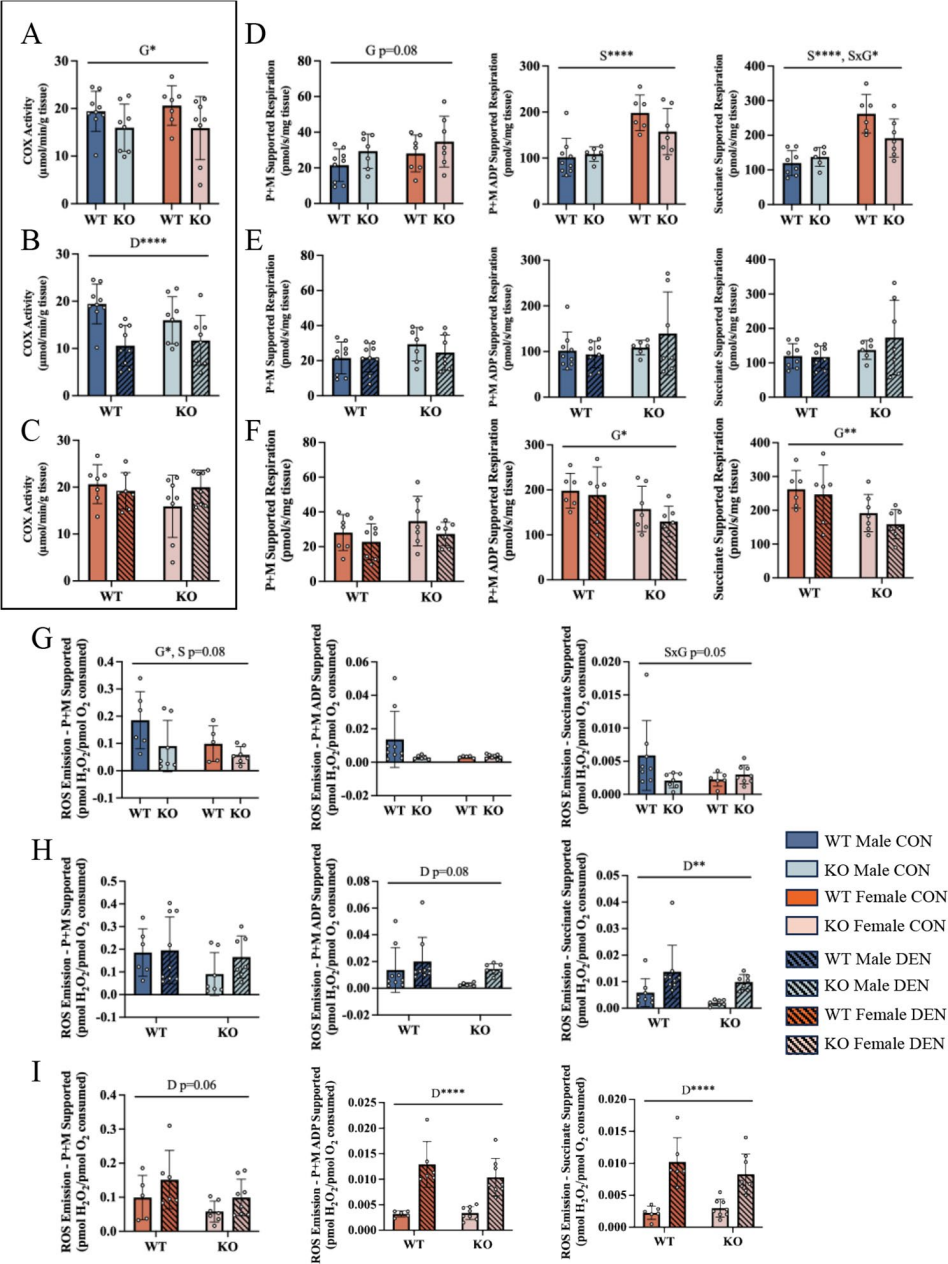
Sex-dependent response in mitochondrial content and function to denervation. Cytochrome c oxidase (COX) activity assessed as a measure of mitochondrial content basally (**A**). COX activity following denervation in male animals (**B**) and female animals (**C**). Oxygen consumption measured in permeabilized muscle fibers in the presence of pyruvate + malate, ADP and succinate in male and female mice basally (**D**). Oxygen consumption in the respective states in male mice following denervation (**E**) and female mice (**F**). ROS emission rates corrected for respiration supported by pyruvate + malate, ADP, and succinate basally in male and female mice (**G**). ROS emission rates under the respective states in male mice following denervation. (**H**) and female mice (**I**). Two-way ANOVAs were conducted with repeated measures between CON and DEN conditions, followed by Tukey post-hoc analyses. P + M, pyruvate malate; G, represents a main effect of genotype; S, denotes a main effect of sex; D, indicates a main effect of denervation; SxG, represents an interaction between genotype and sex; *, *p* < 0.05; **, *p* < 0.01; ****, *p* < 0.0001; *n* = 7

No significant differences in COX activity were observed between WT males and females, however the absence of TFE3 resulted in a 20% decline (Fig. 2A), indicating that basal mitochondrial content is maintained, in part, by TFE3. Using high-resolution respirometry in permeabilized muscle fibers, higher ADP- and succinate-supported oxygen consumption was observed (Fig. 2D) in females in comparison to males, irrespective of genotype. Intriguingly, the absence of TFE3 resulted in a 27% reduction of succinate-supported respiration in females, an observation not found in males (Fig. 2D). Basal ROS emission was lower in KO, compared to WT animals, in a manner that was partly dependent on sex (Fig. 2G) further indicating that TFE3 exerts a differential effect on mitochondrial function in males and females (Fig. 2D and G).

COX enzyme activity was significantly reduced following 7 days of denervation in male animals of both genotypes by 45% in WT, but only by 27% in KO animals (Fig. 2B). Quite surprisingly, COX activity was maintained in both WT and KO females (Fig. 2C), indicating that females are spared against denervation-induced declines in mitochondrial content (Fig. 2C, S1). Mitochondrial respiration across all states was not significantly impacted by denervation (Fig. 2E and F, S1). However, increased ROS emission was observed across all active respiratory states (Fig. 2H and I, S1), with -fold increases being most evident in females (Fig. 2H vs. I). Thus, despite having higher ROS production basally (Fig. 2G), WT males showed the least induction of ROS following chronic denervation. No genotype differences were observed in denervation-induced ROS emission. However, these data suggest that males are partially spared from denervation-induced mitochondrial dysfunction, independent of the presence or absence of TFE3.

**Fig. 3:**
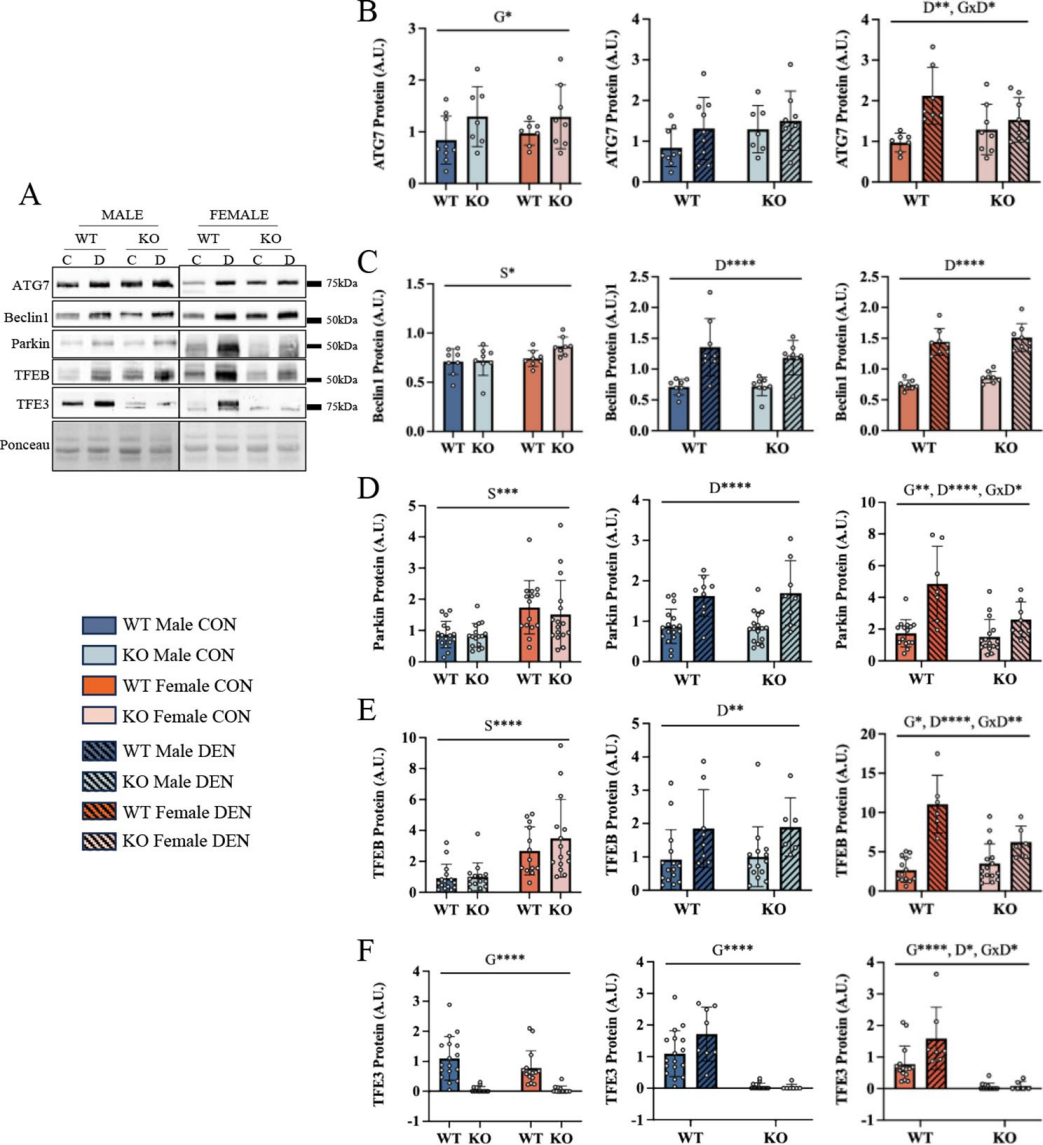
Females exhibit higher autophagy and mitophagy protein levels and these proteins increase dramatically with denervation in a TFE3-dependent manner. Representative blots for autophagy- and mitophagy-related markers following 7 days (7D) of denervation (**A**). ATG7 protein content (**B**) was quantified basally between males and females, then following denervation in male mice and female mice. Similar quantifications were performed for Beclin1 (**C**), Parkin (**D**), TFEB (**E**), and TFE3 (**F**) protein content. Two-way ANOVAs were conducted with repeated measures between CON and DEN conditions, followed by Tukey post-hoc analyses. G, represents a main effect of genotype; S, denotes a main effect of sex; D, indicates a main effect of denervation; GxS, represents an interaction between genotype and sex; GxS, indicates an interaction effect between genotype and sex; GxS, interaction effect between genotype and sex; *, *p* < 0.05; **, *p* < 0.01; ***, *p* < 0.001; ****, *p* < 0.0001; control n-14-16, denervated *n* = 7–8

### Females display profound autophagic and mitophagic protein responses to chronic denervation, and this is blunted in the absence of TFE3

We evaluated mitophagy and autophagy markers basally and in response to 7 days of denervation. Standards were used to make comparisons between males and females, visible in the expanded images (Fig. S4). Elevated levels of TFEB and Parkin as well as the autophagy protein Beclin-1 were observed basally in muscle of female animals (Fig. 3A, C, D and E). A sex difference was not apparent in the autophagy-related marker ATG7, but ATG7 was higher in TFE3 KO animals irrespective of sex (Fig. 3B). Following 7 days of denervation, the upstream autophagy regulator ATG7 was increased by 2-fold only in female WT animals, and this was blunted in the absence of TFE3 (Fig. 3B, S3). In contrast, Beclin1 was increased by ∼1.8-fold in both males and females, irrespective of TFE3 expression (Fig. 3C). Denervation led to marked increases in Parkin protein content in both males and females, an effect that was blunted by the absence of TFE3 in female muscle (Fig. 3D, S3). Denervation also increased TFEB expression by 2.8-fold in WT females. This large increase was significantly blunted in KO female (1.8-fold; Fig. 3E, S3) and male animals. TFE3 was not significantly affected by denervation in in WT mice, and in contrast to TFEB, no sex differences were observed (Fig. 3F).

**Fig. 4:**
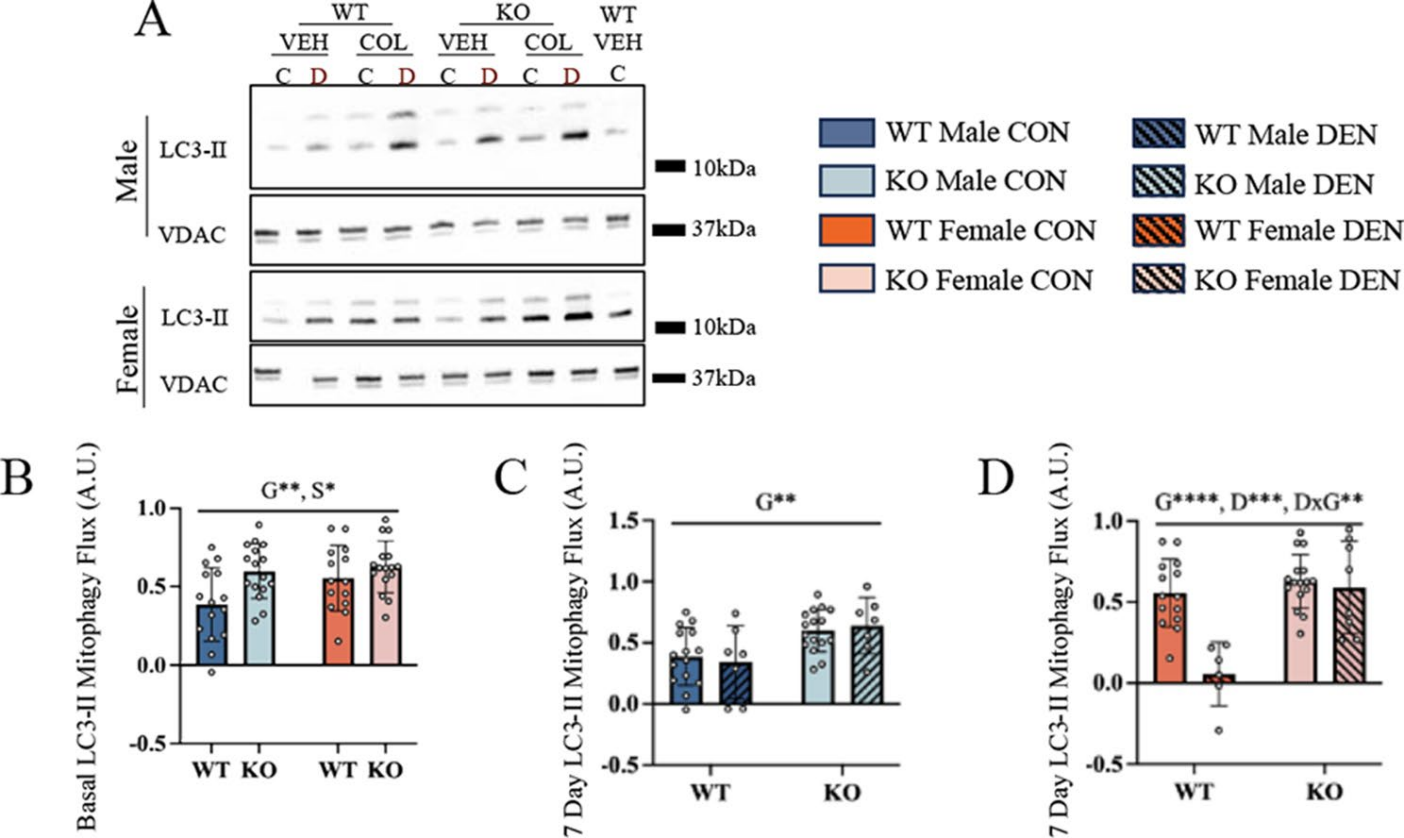
Mitophagic response to chronic denervation is dependent on sex and influenced byTFE3. Representative blots of mitophagy flux following 7 days of denervation (**A**). Quantification of LC3-II mitophagy flux, taken as the difference between colchicine- and vehicle-treated conditions (**A**). LC3-II mitophagic flux was assessed basally in male and female mice (**B**). LC3-II flux was also measured following 7 days of denervation in male mice (**C**) and female mice (**D**). Two-way ANOVAs were conducted with repeated measures between CON and DEN conditions, followed by Tukey post-hoc analyses. G, represents a main effect of genotype; S, denotes a main effect of sex; DxG, interaction effect between denervation and genotype; *, *p* < 0.05; **, *p* < 0.01; ***, *p* < 0.001; control *n* = 14–16, denervated *n* = 7–8

Mitophagy flux was assessed both basally and in response to 7 days of denervation. LC3-II flux was greater in muscle of female animals regardless of genotype (Fig. 4B), corresponding to higher regulatory protein levels (Fig. 3, above). Basal mitophagy flux was also higher in TFE3 KO animals. This genotype-dependent effect was also observed in response to 7 days of denervation, however the changes observed were dependent on sex as WT females decreased their LC3-II mitophagy flux, whereas this was not observed in WT males (Fig. 4C and D). The reduction in mitophagy flux in WT females was attenuated by the absence of TFE3 (Fig. 4D). This reduced mitophagy flux following denervation in WT females presumably supports the maintenance of mitochondrial content, in contrast to the observation in males. However, this inhibition of mitophagy was not evident in females.

lacking TFE3, potentially indicating an impaired ability to mount a sex-appropriate response in the absence of the transcription factor. In KO animals, despite the higher mitophagy flux basally, mitochondrial function is largely similar to that found in WT animals.

### Increases in lysosomal markers in females following denervation are partially dependent on TFE3

**Fig. 5:**
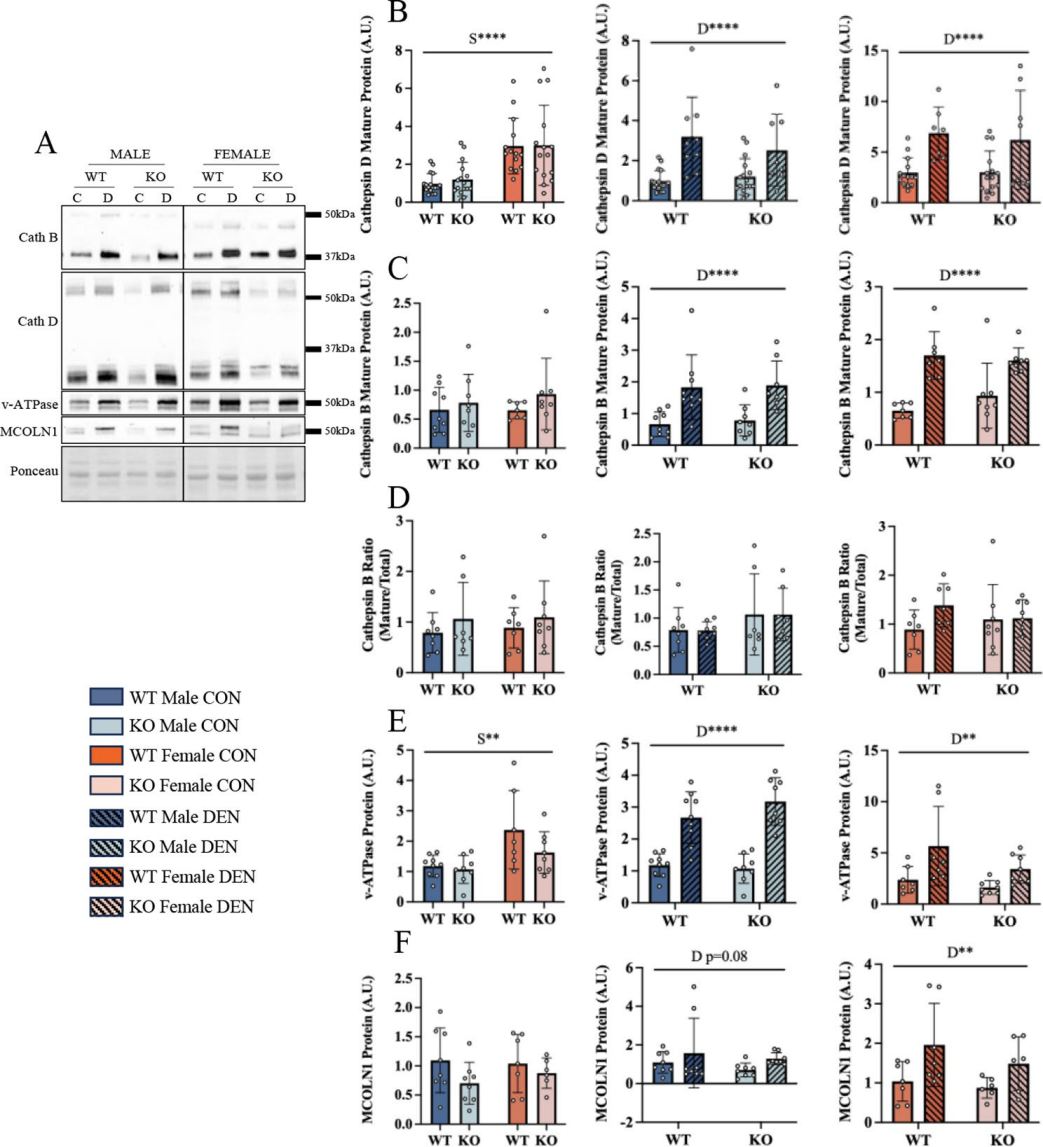
Denervation increases lysosomal markers independent of sex or genotype. Representative blots of lysosomal markers following 7 days of denervation (**A**). Mature Cathepsin D protein content (**B**) was quantified basally between males and females, then following denervation in male mice and female mice. Similar quantifications were performed for mature Cathepsin B (**C**), Cathepsin B ratio (**D**), v-ATPase (**E**), and MCOLN1 (**F**). Two-way ANOVAs were conducted with repeated measures between CON and DEN conditions, followed by Tukey post-hoc analyses. S, denotes a main effect of sex; D, indicates a main effect of denervation; **, *p* < 0.01; ****, *p* < 0.0001; control *n* = 14–16, denervated *n* = 7–8

We measured lysosomal markers basally and following 7 days of denervation. Sex differences were observed for mature Cathepsin D and v-ATPase protein expression, as females had 2- and 1.8-fold higher basal protein expression, respectively (Fig. 5A, B and E). However no differences were observed for Cathepsin B, or MCOLN1 (Fig. 5C and F). Denervation resulted in an induction of lysosomal proteins, as Cathepsin B, Cathepsin D, MCOLN1, LAMP1 and v-ATPase were all upregulated independent of genotype or sex (Fig. 5A-F). No changes were observed in the ratio of mature/total Cathepsin B, which serves as an indication of lysosomal function. Surprisingly, there was no significant impact of the loss of TFE3 on the denervation-induced increases in lysosomal proteins in either sex.

## Discussion

The loss of innervation has severe ramifications for skeletal muscle mass and function. A common feature that has widely been overlooked until very recently is the impact of sex on muscle atrophy. The purpose of the present study was to explore the influence of the transcription factor TFE3 on denervation-induced muscle atrophy between the sexes with a focus on the adaptive roles of lysosomes and mitochondria. Importantly, this transcription factor resides on the X chromosome and thus we speculated that it would be a good candidate to investigate sexual dimorphisms.

### Sex-dependent phenotypes

Females exhibited greater mitochondrial function, characterized by increased oxygen consumption compared to males under basal conditions. Our data also indicate that females have an increased lysosomal content compared to males (Fig. 5). This elevated lysosomal capacity, along with greater mitophagy and autophagy regulatory proteins, could be the reason for the augmented capacity for mitochondrial turnover (i.e. mitophagy flux) that we observed basally in muscle of female animals.

**Fig. 6:**
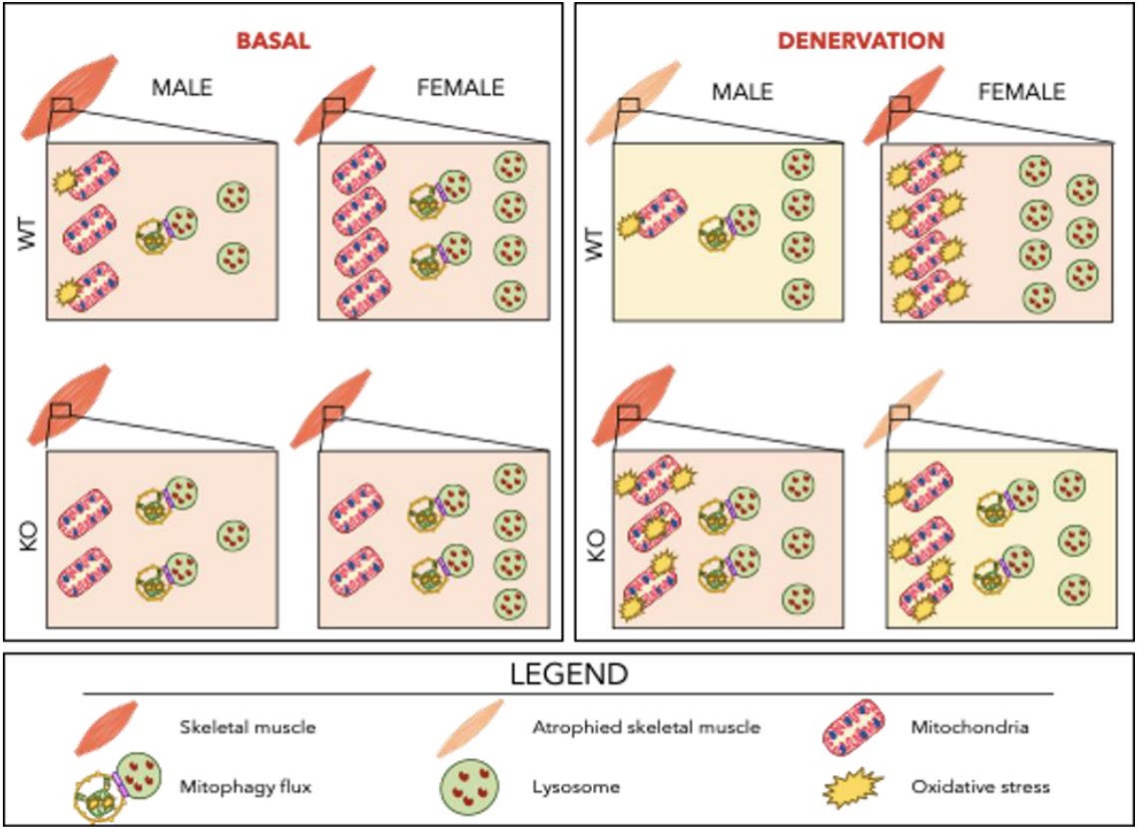
Summary of findings. Basally, females exhibit increased mitochondrial respiration and modestly reduced ROS emission, and this corresponds with heightened mitochondrial turnover supported by greater lysosomal content and higher basal mitophagy flux in comparison to males. Irrespective of sex, the loss of TFE3 results in a modest loss of mitochondrial content but does not impact lysosomal content. In response to 7 days of denervation, wild type males experience significant muscle atrophy and pronounced declines in mitochondrial content with a modest preservation in function. However, wildtype females appear to preserve mitochondrial content at the expense of function likely due to the reduction in denervation-induced mitophagy despite a robust lysosomal induction. The loss of TFE3 in males appears to shift the denervation-induced response towards that of a wildtype female in that mitochondrial content is modestly preserved at the expense of function. In the females, loss of TFE3 reduces the adaptive lysosomal response to denervation and the inhibitory effect on mitophagy is lost

TFE3 has been implicated in autophagy and lysosomal regulation since studies have found that the loss of TFE3 negatively impacts lysosomes under stress conditions [31, 35]. Our results indicate that the lack of TFE3 exerts a subtle metabolic phenotype, as mitochondrial content was reduced in both males and females (Figs. 2 and 6). Intriguingly a partial reduction in mitochondrial respiration was observed in females, but not in males in the absence of TFE3. This indicates that mitochondrial function is regulated in part through TFE3 in a sex-dependent manner. Furthermore, as no differences were observed in lysosomal proteins basally in the absence of TFE3, this cannot be attributed to previously described roles of TFE3 in determining lysosomal content. Moreover, we did not observe any compensation in TFEB at the protein level. This indicates that basal levels of TFEB, along with the possible contribution of other factors such as MITF or FOXO [31, 41–43], are sufficient to maintain lysosomal content basally. Indeed, our recent work using myotubes in culture has confirmed that the loss of both TFE3 and TFEB is required to induce functional and quantitative deficits in lysosomal function and mitochondrial adaptations [44]. Further, contrary to our initial hypothesis, an increase in mitophagy flux was observed in the absence of TFE3 irrespective of sex. This increased turnover potentially supports the reduced mitochondrial content observed in these animals. These data indicate that TFE3 participates in the maintenance of mitochondria in a sex-dependent manner. In addition, we have also recently shown that TFE3 is important for exercise training increases in mitochondrial content [45], suggesting that TFE3 has functions beyond those related to the regulation autophagy / mitophagy pathways.

### Sex-dependent denervation-induced phenotypes

Our data verify that female mice have significantly less muscle mass in comparison to male counterparts. However, females exhibited a preservative effect following 7 days of denervation, as they lost less muscle in comparison to males. This sex difference in atrophy response may be reliant on the model employed, as a divergent time-dependent response was observed between males and females during the progression of hindlimb-unloading-induced atrophy in mice [46]. It is likely that the differences can be attributed to the severity of the model, as denervation generally produces greater losses in muscle mass than those observed with similar periods of hindlimb unloading [1]. In line with this hypothesis, female rats subjected to 14 days of hindlimb unloading experienced a preservation of muscle mass and function in comparison to males [47]. The mechanisms involved may be related to effects of estrogen, since estrogen administration in male rats has been shown to protect against atrophy following 10 days of immobilization, in part attributed to reduced calpain expression [48].

As has been described in numerous accounts, prolonged denervation leads to mitochondrial dysfunction and declines in mitochondrial content [7, 11, 21, 49–51]. Here we show that this response is also sexually dimorphic, such that males lose mitochondrial content while females do not. However, it appears that females are preserving mitochondria at the cost of function, as females exhibited a greater induction of ROS emission with denervation. Thus, the maintenance of mitochondrial content following denervation is likely reflective of an accumulation of dysfunctional mitochondria, rather than a beneficial adaptation. This is in line with previous findings that have also observed a greater elevation in ROS emission in females following hindlimb unloading [18, 19]. Females reduce their mitophagy flux in response to denervation thereby maintaining mitochondrial content, but at the expense of increased dysfunction (Fig. 6). Interestingly, females exhibited a greater induction of ATG7, Parkin and TFEB, indicating that this preference to preserve mitochondria is not due to a lack of lysosomes or autophagy machinery. Thus, these data indicate that males and females have different priorities during the initial phases (1 week) of denervation-induced atrophy, but it remains unclear what drives this dimorphism, and which strategy is beneficial in the long run, when denervation conditions become more prolonged.

We initially hypothesized that in atrophic conditions autophagy and lysosomes would be dysregulated in the absence of TFE3, thereby exacerbating the disuse-induced phenotype. This would be in line with previous reports that have shown that the inhibition of autophagy induces muscle atrophy [52] and can exacerbate muscle wasting [53, 54]. Surprisingly, muscle mass was spared following denervation in the absence of TFE3, an effect that was only observed in males, not females. Further, mitochondrial content was preserved in the absence of TFE3 in females, but similar to their WT counterparts this was accompanied by increased mitochondrial dysfunction (Fig. 6). In response to denervation, mitophagy flux did not decline in the absence of TFE3, leading to the maintenance or increase in mitochondrial content markers with denervation. Thus, our data suggest that TFE3 is playing a sex-dependent role, as the decrease in mitophagy flux observed in WT females was lost in the absence of TFE3.

The induction of autophagy and lysosomal proteins that typically accompany chronic denervation in WT animals was attenuated in the absence of TFE3 in females (Fig. 5). This was matched by a marked reduction in TFEB expression in female mice following 7 days of denervation, and a similar phenomenon was observed in a subset of lysosomal markers including v-ATPase and MCOLN1, both of which are integral to maintenance of lysosomal function [55–57]. However, while the denervation-induced expression of these lysosomal markers was not as great in males, the absence of TFE3 had no impact. Moreover, the denervation-induced elevation in ATG7 and Parkin were severely blunted in females lacking TFE3. Thus, our data suggest that TFE3 is dispensable basally, as well as in mediating denervation-induced increases in lysosomal and autophagy-related proteins in males. However, TFE3 appears to be more critical in the lysosomal and autophagy responses in females, indicating a sexually dimorphic function of TFE3 in response to denervation-induced muscle atrophy of this relatively short-term duration.

## Overall conclusion

Our findings add to the emerging literature supporting a sexually dimorphic response of muscle to denervation, as well as identify an important function for TFE3 in determining this divergent response (Fig. 6). Females have a tendency to preserve muscle mass and mitochondria in response to denervation, perhaps at the expense of function by reducing mitochondrial clearance through mitophagy. This is not due to a limitation on lysosomes or autophagy machinery, as females exhibit a greater expression of lysosomal and autophagy proteins. On the other hand, males preferentially maintain mitochondrial function at the expense of content by maintaining high levels of mitophagy during denervation to help clear dysfunctional organelles. It appears that despite having the lysosomal and autophagic capacity, females do not rely on these mechanisms to preserve muscle mass following 7 days of denervation. Since females exhibited a decrease in mitophagy following denervation, this likely contributed to an accumulation of dysfunctional organelles. Despite this, females exhibited a modest sparing of muscle mass. It should be noted that the present study aimed to understand and describe the lysosomal and mitophagic responses to denervation, however it is widely understood that these are not the sole mechanisms at play during muscle atrophy. Recent work from others has begun to explore the impact of sex on the ubiquitin proteasome system and protein synthesis during atrophy [19, 46, 47], however this remains mostly uncharacterized. Our data underscore the value of further exploring the molecular underpinnings that regulate how males and females respond to denervation and solidify the need to develop sex-specific strategies to combat muscle atrophy.

### Electronic supplementary material

Below is the link to the electronic supplementary material.

Supplementary Material 1

## Data Availability

The datasets used and analysed during the current study are available from the corresponding author on reasonable request.

## Abbreviations

ADP: Adenosine diphosphate
ATP: Adenosine triphosphate
ATG7: Autophagy related 7
COL: Colchicine
COX: Cytochrome c oxidase
COX I: Cytochrome c oxidase subunit 1
COX IV: Cytochrome c oxidase subunit IV
CTSD: Cathepsin D
EDL: Extensor digitorum longus
KO: Knockout
LAMP1/2: Lysosome membrane associated protein 1/2
LC3: Microtubule-associated protein 1 A/1B light chain 3B
MCOLN1/TRPML1: Mucolipin1
MiT: Microphthalmia
MQC: Mitochondrial quality control
ROS: Reactive oxygen species
SOL: Soleus
SQSTM1 or p62: Squestosome 1
TA: Tibialis Anterior
TFEB: Transcription factor EB
UQCRC2: Ubiquinol-Cytochrome c reductase core protein 2
v-ATPase: Vacuolar-type ATPase
VDAC: Voltage-dependent anion channel
VEH: Vehicle
WT: Wildtype
