# Supplementary material for: Dimorphic effect of TFE3 in determining mitochondrial and lysosomal content in muscle following denervation
Source: Skelet Muscle. 2024 Apr 20;14:7. doi: 10.1186/s13395-024-00339-1 (PMC11031958; doi:10.1186/s13395-024-00339-1)
Supplement: Supplementary file 1 — Supplementary Material 1 [file 13395_2024_339_MOESM1_ESM.docx]

**Supplemental Figures:**


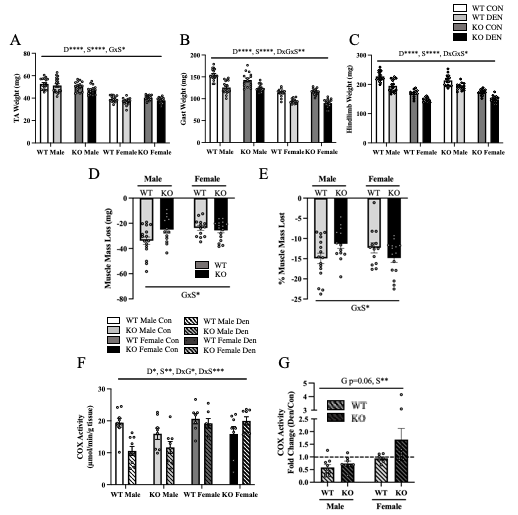


**Supplemental Fig. 1:** Expanded analyses of muscle mass and COX activity. Muscle mass lost following 7 days of denervation, 3-way ANOVAs were conducted to examine the effect of genotype, sex and denervation (A-C). The total loss of hindlimb muscle weight was also presented as an absolute (D) and as a percent, relative to control limb (E) based on the data presented in Fig. 1I, 1J (n = 16). Based on the COX activity data presented in Fig. 2D, a 3-way ANOVA was performed to examine the effects of genotype, sex and denervation (F), and is also depicted as a fold change for simplicity (G). Two-way ANOVAs were conducted on the fold changes, followed by Tukey post-hoc analyses (also done for 3-way ANOVA). G, represents a main effect of genotype; S, denotes a main effect of sex; D, indicates a main effect of denervation; GxS, represents an interaction between genotype and sex; DxG, signifies an interaction between denervation and genotype; DxGxS, denotes a 3-way interaction effect; *, p < 0.05; **, p < 0.01; ***, p < 0.001; ****, p < 0.0001, n = 7–8.


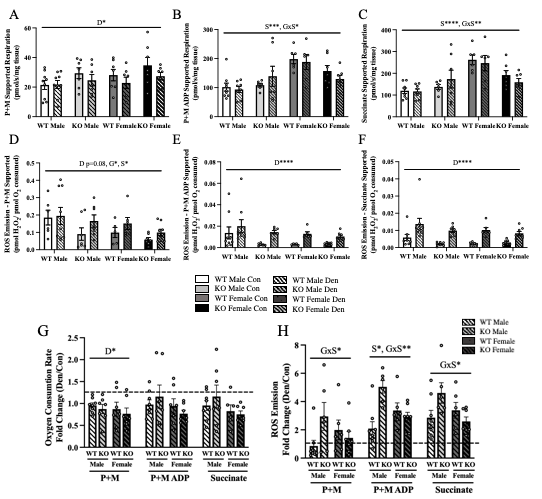


**Supplemental Fig. 2:** Expanded analyses of oxygen consumption and ROS emissions. Based on data presented in Fig. 2D-F a 3-way ANOVA was conducted to assess the effect of genotype, sex and denervation during P + M-supported and active respiratory states (A-C). The same was done for the corresponding ROS emission data presented in Fig. 2G-I (D-F). This also depicted as a fold change for simplicity (oxygen consumption, G; ROS emission, H). Two-way ANOVAs were conducted on the fold changes, followed by Tukey post-hoc analyses (also performed following 3-way ANOVA). P, pyruvate; M, malate; D, indicates a main effect of denervation; G, represents a main effect of genotype; S, denotes a main effect of sex; GxS, represents an interaction between genotype and sex; *, p < 0.05; **, p < 0.01; ***, p < 0.001; ****, p < 0.0001, n = 7–8.


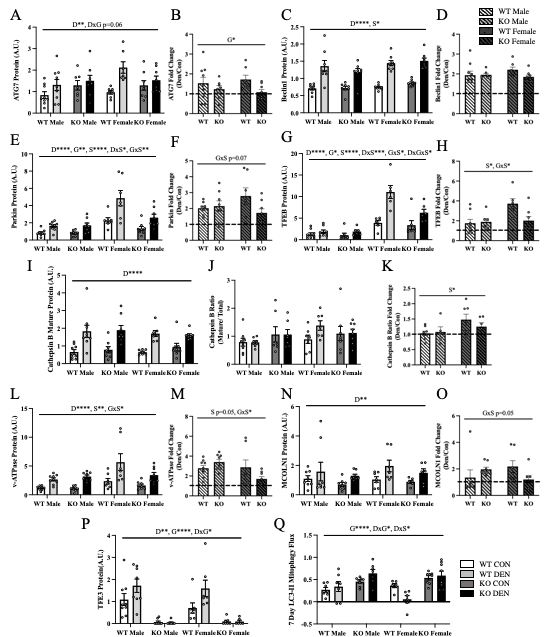
**Supplemental Fig. 3:** Expanded analyses of protein content changes following 7-days of denervation.

A 3-way ANOVA was conducted on ATG7 protein content changes following 7 days of denervation based on data presented in Fig. 5A (A), and also represented as a fold change compared to control (B). Similar analyses were also done for Beclin1 (C, D; based on Fig. 5B), Parkin (E, F; based on Fig. 5C), TFEB (G, H; based on Fig. 5C), Mature Cathepsin B (I) and its ratio (J, K; based on Fig. 7C), v-ATPase (L, M; based on Fig. 7E) and MCOLN1 (N, O; based on Fig. 7F). Two-way ANOVAs were conducted on the fold-changes, and Tukey post-hoc analyses were done for all. D, indicates a main effect of denervation; G, represents a main effect of genotype; S, denotes a main effect of sex; DxG, denotes an interaction effect between denervation and genotype; DxS, signifies an interaction effect between denervation and sex; GxS, represents an interaction between genotype and sex; DxGxS, indicates a 3-way interaction; *, p < 0.05; **, p < 0.01; ***, p < 0.001; ****, p < 0.0001, n = 7–8.


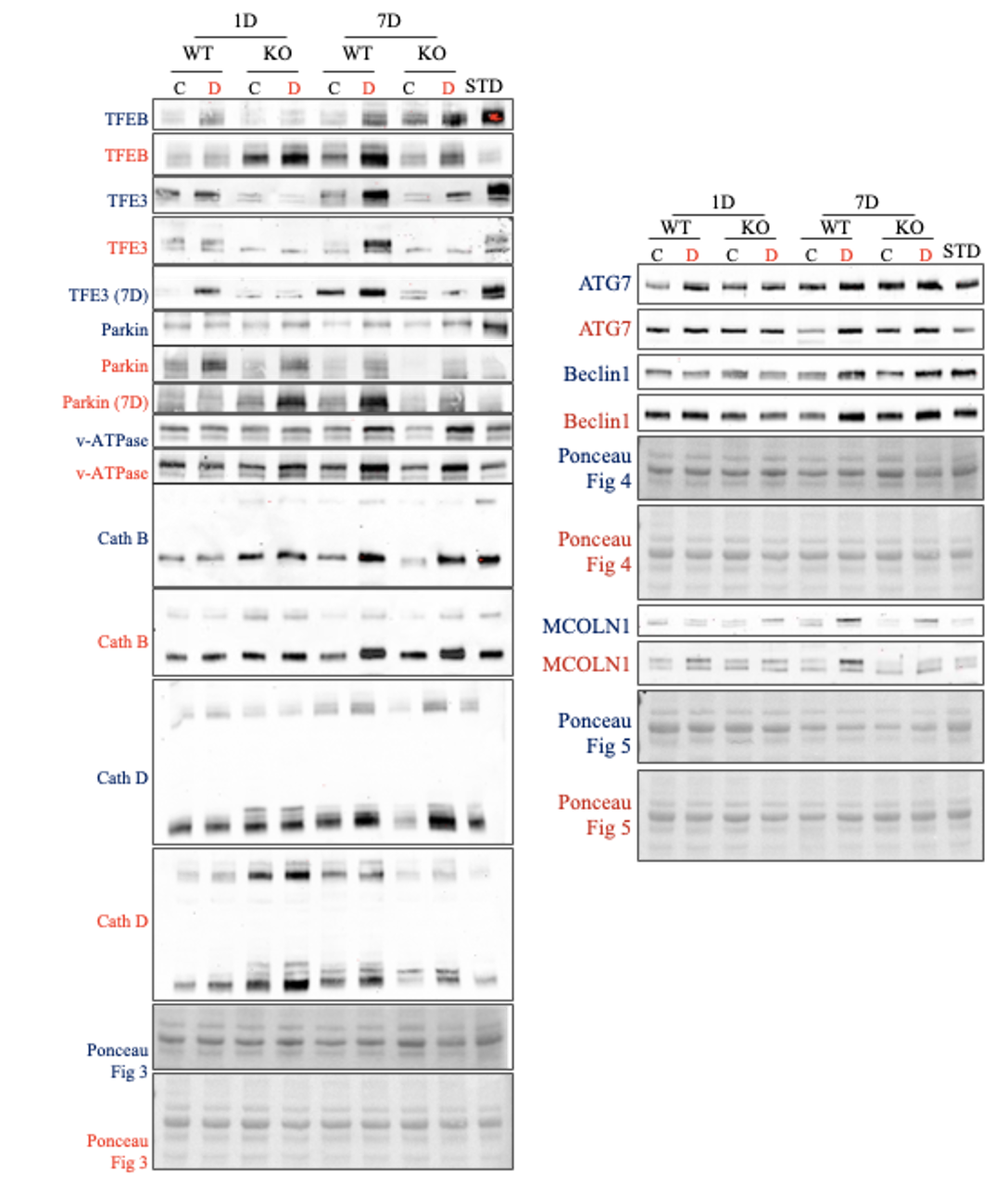
**Supplemental Fig. 4:** Full western blots expanded. Full blots from Figs. 3, 4, 5, including the standard that was used for quantification. 1Day (1D) denervation data are also presented in the blots, but not further discussed in the manuscript.
